# A systematic review of diet and medication use among centenarians and near-centenarians worldwide

**DOI:** 10.1007/s11357-024-01247-4

**Published:** 2024-07-05

**Authors:** Zhaoli Dai, Sue Yi Lee, Srishti Sharma, Shahid Ullah, Edwin C. K. Tan, Henry Brodaty, Aletta E. Schutte, Perminder S. Sachdev

**Affiliations:** 1 School of Population Health, Faculty of Medicine and Health, University of New South Wales (UNSW Sydney), Sydney, NSW 2052 Australia; 2 School of Pharmacy, Faculty of Medicine and Health, The University of Sydney, Sydney, NSW 2006 Australia; 3 College of Medicine and Public Health, Flinders University, Bedford Park, Adelaide, SA 5042 Australia; 4 Centre for Healthy Brain Ageing, Discipline of Psychiatry and Mental Health, Faculty of Medicine and Health, University of New South Wales (UNSW Sydney), Sydney, NSW Australia; 5 The George Institute for Global Health, University of New South Wales (UNSW Sydney), Sydney, NSW 2052 Australia; 6 UNSW Ageing Futures Institute, University of New South Wales (UNSW Sydney), Sydney, NSW Australia

**Keywords:** Longevity, Centenarian, Diet, Nutrition, Medication, Lifestyle

## Abstract

Centenarians represent a phenomenon of successful aging. This systematic review aimed to understand lifestyles and health practices, focusing on diet and medication use for healthy longevity in community-based adults 95 years or over. Medline, CINAHL, Scopus, and gray literature were searched from 1 January 2000 to 10 December 2022. Study quality was assessed using the Modified Newcastle-Ottawa Scale (mNOS). Pooled prevalence [%; 95% confidence interval] for categorical variables and pooled mean for continuous variables were estimated for demographics, weight status, lifestyle factors, medications, and health conditions. Of 3392 records screened, 34 studies were included in the review, and 71% (24/34) met the 6/8 criteria in mNOS. Centenarians/near-centenarians’ ages ranged from 95 to 118 years, with 75% (71–78%) female and 78% (68–88%) living in rural areas. They had an overall healthy lifestyle: current smoking (7%; 5–9%), drinking (23%; 17–30%), normal weight (52%; 42–61%), overweight (14%; 8–20%), physical activity (23%; 20–26%), and sleep satisfaction (68%; 65–72%). Diet averaged 59.6% carbohydrate, 18.5% protein, and 29.3% fat; over 60% consumed a diverse diet, and < 20% preferred salty food, contributing to lower mortality risks and functional decline. About half used antihypertensives (49%; 14–84%) or other cardiovascular drugs (48%; 24–71%), with an average of 4.6 medications. Common health issues included impaired basic activities of daily living (54%; 33–74%), hypertension (43%; 21–65%), and dementia (41%; 23–59%). The findings of this systemic review underscore the pivotal role of dietary practice and weight management in healthcare strategies to promote healthy ageing. It also recognises rural living styles and sleep hygiene as potential factors contributing to healthy longevity.

**Supplementary Information:**

The online version contains supplementary material available at 10.1007/s11357-024-01247-4.

## Introduction

Population ageing has become a global challenge [1]. This demographic shift is driven by declining fertility rates and increasing life expectancy, particularly among high- and mid-income countries. The implications of a growing older population include reduced labour productivity and increased social burdens and healthcare expenses, primarily due to older adults often experiencing reduced functional independence, disability and multiple chronic conditions. Therefore, it is crucial to develop strategies that enhance disease management, prevent disability and promote well-being to ensure the quality of life of the golden years. Proper nutrition and medication use are vital to achieving these goals [2–5].

Centenarians represent a remarkable phenomenon of successful ageing. They often have lower disease prevalence or delayed onset of chronic conditions than the general older population [6, 7]. Previous studies have suggested that centenarians experience fewer chronic diseases and maintain their independence in daily life well into their 90s [6, 8–10]. While genetic factors play a role in extreme longevity, non-genetic or environmental factors have been estimated to account for over 60% of successful ageing [11, 12]. Additionally, lifestyle and environmental factors may interact with genetic, epigenetic and phenotypic factors to affect longevity [11, 13].

There has been a substantial increase in centenarians globally, rising from 151,000 in 2000 to 573,000 in 2021—a four-fold increase, with projections of 3.5 million centenarians by 2050. While most centenarians live in countries with political and economic stability, which provide better access to healthcare, medications, treatment, nutrition, and housing, the number of centenarians varies by country, regardless of economic development status [14]. This indicates that cultural practices in lifestyles can play a pivotal role.

To our knowledge, no comprehensive reviews are available to systematically examine centenarians’ lifestyles and health practices, such as dietary habits (including diet patterns, food groups and supplements) and common medication use concerning ageing health outcomes. In this systematic review, our research questions centred on the concept of “food as medicine” [15] and the potential adverse effects of polypharmacy, defined as five or more medications [16] on age-related health outcomes [17, 18], to understand lifestyles and health practices among centenarians and near-centenarians.

## Methods

The protocol of this systematic review was registered and published in the Open Science Framework in January 2023 [19].

### Data sources and searches

We modified the search strategy used in a previous systematic review among near-centenarians and centenarians [20] to search for peer-reviewed journal articles in MEDLINE (via OVID), CINAHL (via EBSCO), Scopus and grey literature. We restricted the search among articles published in English from 1 January 2000 to 10 December 2022.

The systematic review search was conducted on 10 December 2022 (ZD), with centenarian, oldest-old, 100 years old and over, longevity, healthy ageing and successful ageing in the keywords. We identified articles that reported the oldest old’s diet, nutrition and/or medication use. The search strategy used to retrieve the studies is described in Supplementary materials (S1. Search strategies). Additionally, we hand-searched articles based on the references of relevant studies to identify if there were any missing ones.

### Inclusion and exclusion criteria

Details of the inclusion and exclusion criteria were previously published [19] and are summarised in Supplementary Table s1. Briefly, epidemiological studies conducted among centenarians (aged 100+) or those with a mean/median age of 95 or above were included. The reason for including near-centenarians was that some studies combined near-centenarians and centenarians as a cohort to study extreme longevity.

### Data screening and selection

Two reviewers (SL and SS) independently screened the titles and abstracts of all retrieved records after removing duplicates from the databases, followed by reviewing the full-text articles of potential studies to identify eligible studies based on the inclusion and exclusion criteria. This process was completed using the automatic screening tool via Covidence (https://www.covidence.org). Any discrepancies regarding the screening or selection of the studies were resolved through discussion to reach a consensus with a third reviewer (ZD).

### Quality of study assessment (risk of bias assessment)

To assess study quality, we used the Modified Newcastle-Ottawa Scale (mNOS) for cross-sectional and longitudinal studies [21] on domains including representation of the study population, attrition, exposure measures, outcome measures, confounders, statistical adjustment, funding source and authors’ declaration of conflicts of interest.

### Data extraction

Two reviewers (SL and SS) independently extracted the data from the eligible studies using an electronic data capture tool via Covidence. The third reviewer (ZD) reviewed and compared the extraction. Discrepancies were resolved through discussions among the reviewers to reach consensus.

### Basic information

Article information (title, lead author and published year), the country where the study was conducted, study design, follow-up time in longitudinal studies, sample size, number of centenarians/near-centenarians, identification method for centenarians, exposures of interest and outcomes (Supplementary Table s2).

### Baseline characteristics of centenarians/near-centenarians

Number of centenarians/near centenarians, age, sex, level of education, living arrangement, urban or rural living, smoking status, alcohol drinking status, physical activity, and sleep quality.

### Exposures of interest

Dietary patterns, food groups, dietary habits, nutrients, and weight status based on body mass index (BMI) were the primary exposures of interest. We also extracted macronutrient intake, serum albumin and lipid profiles. For medicines, we extracted the type and number of the medications described in the articles.

### Outcome variables

These include any ageing outcomes or conditions reported cross-sectionally or longitudinally. We extracted the adjusted risk ratios to estimate the exposures mentioned above in association with a health outcome, such as odds ratios (ORs) or hazard ratios (HRs) and the corresponding 95% confidence intervals (CIs). If studies did not include adjustments for confounders in regression analysis, they were excluded from the evidence synthesis.

### Data analysis

We conducted pooled analyses for categorical variables to estimate the prevalence of demographic characteristics and lifestyle factors if at least two studies reported the data, using the metaprop package [22] in Stata (version 16) to estimate the mean prevalence and its respective 95% CI using a random-effects model. Forest plots were also plotted. For continuous variables such as macronutrient distribution, serum levels of albumin and lipid profiles, and number of mediations, we used this formula [∑ {mean_1-i_ X n_1-i}_/∑ n_1-I_] to calculate the weighted mean.

Due to the variability of dietary or medication exposures and ageing outcomes, i.e. less than two studies assessed the same associations, we could not conduct a meta-analysis. Instead, we narratively summarised these results. Sensitivity analysis was performed for the prevalence of a characteristic by removing studies with over 90% of the representation (this was done for the characteristic of  the prevalence of living in rural areas in this review).

## Results

**Fig. 1:**
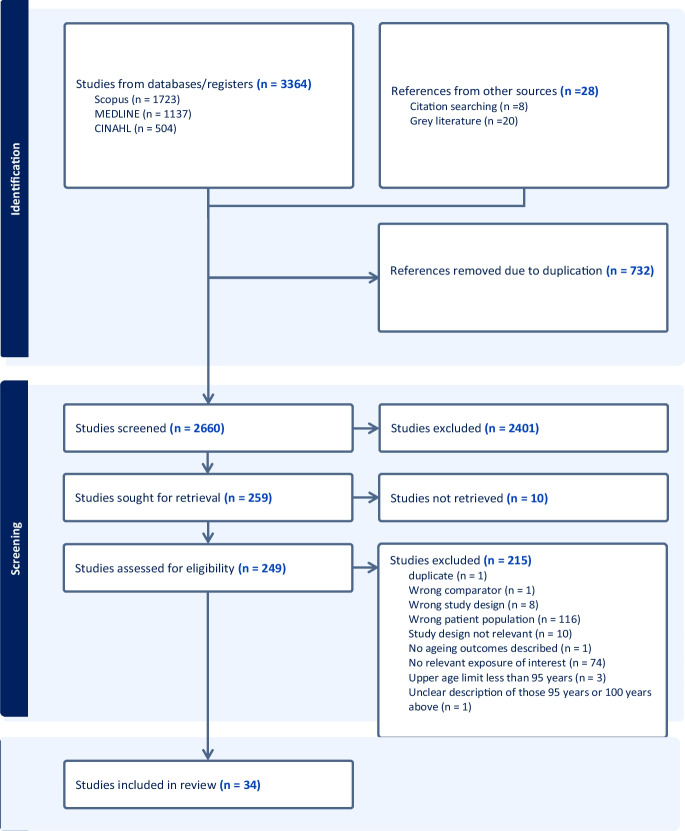
PRISMA flowchart of study selection

Among the 34 studies included (Fig. 1. PRISMA flow chart), 16 were among Asian populations, conducted in China (*n* = 15) and Japan (*n* = 1); one from Australia, and the rest were based in Europe (*n* = 17)—nine in Italy, three in Greece and one each from Germany, Denmark, Sweden, France and Portugal. The number of centenarians or near-centenarians ranged from 16 to 8,908, with a median of 162 (Supplementary Table s2).

The quality of the studies is assessed in Table 1 (the assessment of each study can be found in Supplementary Table s3). Of the studies included, 71% (24/34) met 6/8 of the criteria. However, half of the studies did not mention potential confounders (50%) or adjust for confounders in regression analysis (47%).

**Table 1:** Study quality assessment based on “The Newcastle Ottawa Scale (NOS) for assessing the quality of nonrandomised studies in meta-analysis” among the 34 included studies

Domain	Number of studies met the criterion	% studies met the criterion
1. Are all study groups derived from similar source/reference populations?	31 (yes)	91
2. Is attrition significantly different across study groups?	31 (no)	91
3. Is the measure of exposure valid?*	26 (yes)	76
4. Is the measure of outcome valid?**	27 (yes)	79
5. Did the authors disclose a conflict of interest?	23 (22 without conflict of interest; 1 with conflict of interest)	68
6. Did the study identify potential confounders?	17 (yes)	50
7. Did the study have statistical adjustment?	16 (yes)	47
8. Did the study disclose the funding source?	24 (yes)	71

^*^Valid measures of exposure were defined as blood biomarkers or objective measures by a valid tool or instrument rather than self-report

^**^Valid measures of outcomes were referred to hospital/registry records or doctor’s diagnosis

The pooled prevalence of demographic characteristics and non-dietary lifestyle factors are summarised in Table 2: 75% were females, 88% had education below high school, over half lived with others (57%) and 16% lived in nursing facilities; most lived in rural areas (78%) or did not smoke, with 7% as current smokers and 16% as former smokers. Alcohol consumption was moderately low, with daily drinkers at 23% and 11% as former drinkers. Less than a quarter were physically active. In two studies that surveyed sleep satisfaction [23, 24], the pooled prevalence was 68%. The forest plots for these estimated proportions are in Supplementary Figure s1- Figure s8.

**Table 2:** Summary of centenarians’ characteristics in the included studies

Characteristics	Number of studies that provided the information	Pooled prevalence (%) (95% confidence interval)
Female centenarians	28	75 (71, 78)
Level of education		
Below high school	9	88 (82, 92)
High school to associate degree	3	10 (0.4, 20)
Living arrangement		
Alone	7	10 (5, 14)
Living with others	6	57 (26, 88)
Living in nursing facilities	6	16 (8, 24)
Regionality		
Urban	5	37 (0, 90)
Rural	6	78 (68, 88)
Smoking status		
Former	4	16 (12, 19)
Current	12	7 (5, 9)
Alcohol drinking status		
Former	3	11 (6, 16)
Daily	10	23 (17, 30)
Physically active	3	23 (20, 26)
Sleep satisfaction	2	68 (65, 72)

The summary of body weight status by BMI, diet quality, food group consumption and serum levels of albumin and lipid profiles are described in Table 3.

**Table 3:** Prevalence of body weight status, diet quality or habits and nutritional indicators among centenarians or near centenarians in the included studies

**Body weight and nutrition status**	**Number of studies**	**Pooled prevalence (%) (95% confidence interval)**
Body weight status		
Underweight	7	33 (14, 52)
Normal weight	7	52 (42, 61)
Overweight	6	14 (8, 20)
Obese	4	4 (0.4, 7)
**Nutritional factors**	**Studies specified**	**Pooled estimated mean**
Macronutrient composition	Hao et al. 2019 (all three macronutrients) [23], Cai et al. 2022 (all three macronutrients [25]), and Fastame et al. 2022 (carbohydrate and protein only) [26]	Percent carbohydrate/total energy intake: 59.6% Percent protein/total energy intake: 18.5% Percent fat/total energy intake: 29.3%
**Diet quality and food groups**	**Studies specified**	**Summary of study results**
Diet quality	Li et al. 2021 [27]	DDS: 23/38 (61%) in high DDS scores (5–9)
Dietary habits	Wu et al. 2017 [28]	65% considered they had good dietary habits (defined as eating a variety of food groups, including staple foods, fruit and vegetables and protein-rich foods)
Fruit and vegetables	Stathakos et al. 2005 [29]	Consumed vegetables daily
Poultry, fish or legumes	Stathakos et al. 2005 [29]	Poultry, fish and legumes 2–3 times weekly; and rarely consumed red meat
Salty/smoked food	Hao et al. 2019 [23]	73.5% preferred plain food; 18.8% preferred slightly salty food; 4% preferred slightly spicy food; 9.9% preferred sweet food
	Wu et al. 2017 [28]	11% avoided sweet or fatty food and 4.8% preferred salty food
	Li et al. 2021 [27]	Mean intake of sodium per day: 1648.2 mg
	Zhang et al. 2020 [30]	70% consumed smoked and pickled foods, preferred high-salt food and drank milk, and 86% consumed eggs
**Other metabolic markers**	**Studies specified**	**Pooled estimated mean**
Level of albumin (g/dL)	Basile et al. 2003 [31]; Croize-Pourcelet et al. 2022 [32]; Fu et al. 2020 [33]; Savarino et al. 2001 [34]	3.8 g/dL (reference range: 3.5–5.5 g/dL) [35]
Total triglyceride (mg/dL)	Bucci et al. [36]; Fu et al. [33]; Hai et al. [37]; Li et al. [27]; Montesanto et al. [38]; Wong et al. [39]	111 mg/dL (reference range: < 150 mg/dL) [40]
Total cholesterol (mg/dL)	Bucci et al.2014 [36]; Hai et al. 2022 [37]; Montesanto et al. 2019 [38]; Wong et al. 2019 [39]	188.3 mg/dL (reference range: < 200 mg/dL) [35]
LDL cholesterol (mg/dL)	Fu et al. 2020 [33]; Hai et al. 2022 [37]; Montesano et al. 2019 [38]; Wong et al. 2019 [39]	109 (reference range: < 100 mg/dL) [35]
HDL cholesterol (mg/dL)	Fu et al. 2020 [33]; Hai et al. 2022 [37]; Montesanto et al. [38] Wong et al. 2019 [39]	54.4 (reference range: male: > 40; female: > 50 mg/dL) [35]

*DDS*, dietary diversity score; *LDL*, low-density lipoproteins; *HDL*, high-density lipoproteins

### Weight status

Over half (52%) were in the normal weight category, 33% underweight and 14% overweight. The prevalence of obesity was low, with a pooled estimate of 4% (Supplementary Figure s9).

### Overall diet

Among the ten studies that mentioned overall diet and dietary habits, three estimated macronutrient composition [23, 25, 26], indicating the average intake of carbohydrates being 59.6% of the total energy intake (range: 57–65%); the average protein intake was 18.5% (range: 12%–32%) and the average fat intake was 29.3% (range: 27–31%).

One Chinese study reported diet quality using the dietary diversity score (DDS) and reported that 61% of the 38 centenarians versus 54% of the comparison group (250 offspring or spouses) were in the high DDS category (scored 5–9) [27]. Other single studies mentioned centenarians’ healthy dietary habits: one study reported that 65% of the 564 Chinese centenarians had good dietary habits; they consumed various foods such as staple foods, fruit and vegetables, and protein-rich foods [28]. Another study reported that a cohort of 489 Greek centenarians consumed healthy food, such as olive oil, dairy products and vegetables daily; they also consumed poultry, fish and legumes 2–3 times weekly but rarely consumed red meat [29]. Overall, centenarians or near-centenarians consumed a healthy diet [23, 27, 28, 30]; two out of four Chinese studies indicated that 70% of the centenarians preferred salty foods or had a mean daily intake of sodium of 1,648 mg [27, 30] (Table 3).

Regarding supplement use, no studies reported these intakes.

### Other metabolic markers

#### Albumin

Albumin is the most abundant circulating protein found in plasma, representing half of the total protein content (3.5 to 5 g/dL) of plasma in healthy humans [35]. Several studies measured centenarians’ serum levels, ranging from 3.5 to 3.9 g/dL [31–34]. The pooled estimated mean suggests a normal level of 3.8 g/dL.

### Lipid profiles

#### Total triglycerides

Six studies measured serum total triglycerides. The mean/median level ranged from 90.3 to 124 mg/dL, with an estimated mean of 111 mg/dL, indicating a normal range of total triglycerides (reference range: < 150 mg/dL) [40].

#### Total cholesterol, LDL cholesterol and HDL cholesterol

The range of mean/median of total cholesterol [36–39, 41] was 178 to 196 mg/dL. The estimated pooled average was 188.3 mg/dL, indicating a normal range (< 200 mg/dL) [35]. Similarly, the estimated mean of HDL cholesterol of 54.4 mg/dL in the included studies suggested a healthy level. However, the estimated pooled average of LDL cholesterol of 109 mg/dL suggested a slightly higher value than the reference range (< 100 mg/dL) [35].

## Medication use

### Medication type

Several studies summarised medication use among the oldest old. Among the nine types of medications, the two most common ones were those for lowering blood pressure, including diuretics [36, 37, 42, 43] and other medications for cardiovascular diseases [36, 42–44] (Table 4). Other drug use included 40.9% [32] and 29% [43] for sleeping pills; 15.6% [44] and 50% [32] for psychotropics; 6.0% for respiratory drugs [44], 25% for lipid-lowering medications at [39] and 10.6% for therapies for orthopaedic conditions [44]. The forest plots can be found in Supplementary Figure s10.

**Table 4:** Pooled prevalence of medication use and health conditions among centenarians in the systematic review

**Medications**	**Number of studies**	**Pooled prevalence, % (95% confidence interval)**
Antihypertensive medications	4	49 (14, 84)
Cardiovascular drugs	4	48 (24, 71)
Number of medications	4	Mean: 4.6 (range: 0–12)
**Health conditions**	**Number of studies**	**Pooled prevalence, % (95% confidence interval)**
Activities of daily living ( impairment	6	54 (33, 74)
Hypertension	5	43 (21, 65)
Dementia or cognitive impairment	3	41 (23, 59)
Type 2 diabetes	3	22 (0, 52)
**Health conditions**	**Studies specified**	**Narrative results (%)**
Anaemia	Lv et al. [45]	49
Anxiety	Li et al. [27]	5
Chronic kidney disease	Lv et al. [45]	75
Circulatory system disorder	Hagberg et al. [46]	39
Cognitive impairment	Croize-Pourcelet et al. [32]	46
Congestive heart failure	Schmidt et al. [42]	29
Depression	Croize-Pourcelet et al. [32]	46
Dyslipidaemia	Hai et al. [37]	29
Frailty	Bucci et al. [36]	58
Frequency of hospitalization	Hao et al. [47]	0: 69.4; 1–2: 18.7; >=2: 12.1
Good hearing and vision (combined)	Hagberg et al. [46]	20
Hearing impairment	He et al. [48]	31
Hip fracture	Hagberg et al. [46]	11–39
Incontinence	Croize-Pourcelet et al. [32] Andersen-Ranberg et al. [43]	50; 60
Osteoarthritis of knee, hip, shoulder and spine	Andersen-Ranberg et al. [43]	54
Renal failure	Croize-Pourcelet et al. [32]	74
Vision impairment	He et al. [48]	28

### Number of medications

Four studies specified the number of medications used among centenarians, with a pooled mean of 4.6 medications ranging from 0 to 12. One study [49] was excluded from this analysis, as the number of medications (mean: 2.1) was counted among centenarians and women in other age groups. One study, including 207 healthy centenarians, provided the median (IQR) for medications as 3 (2, 5) [43], and the rest reported an average of 5 or more medications [32, 36, 44].

### Health conditions

There were 25 conditions mentioned in the included studies, ranging from anaemia, anxiety and chronic kidney disease to a disability, type 2 diabetes, hypertension and vision impairment (Table 4). In a pooled analysis, over half of the centenarians had impairment in basic Activities of Daily Living (ADL) (54%). Fewer than half had hypertension (43%), dementia or cognitive impairment (41%), and diabetes (22%). Other conditions were narratively summarised based on single study reports (Table 4). Supplementary Figure s11 provides the forest plots for the pooled prevalence of these conditions.

### Association between diet/nutrition and ageing health outcomes

Due to the high heterogeneity of the analyses, no meta-analysis could be conducted between diet/nutrition and an ageing outcome; furthermore, no studies specifically examined the relationship between drug use and health outcomes. Hence, we summarised the results based on the regression analyses for the relationships between diet or nutrition, other lifestyles and non-lifestyle factors, and an ageing outcome in Table 5. The evidence suggests that good dietary habits, defined by consuming various types of food, including staple food, vegetables and/or fruit, and protein-rich food [28], or a higher DDS [27, 50] was associated with a lower risk of ADL impairment or mortality, while a lower geriatric nutrition risk index [41] or malnutrition [38] was significantly associated with a lower likelihood of reaching longevity (100 yr+) or increased mortality risk. Among centenarians, the strongest associations were observed for a higher versus a lower DDS that reduced mortality risk by 44% [those scored 6 vs. those scored 2: adjusted OR: 0.56; 95% CI: 0.53, 0.58] [50]. By contrast, a low (< 5) versus high (> 7) DDS increased over twofold the odds of having depression [adjusted OR, 2.24; 95% CI, 1.01–4.96] among centenarians and their offspring and spouses [27]. Furthermore, salt preference was a significant risk factor for basic ADL impairment [28]: compared to those without salt preference, centenarians who consumed a higher salt intake had over a 3.6-fold risk of experiencing basic ADL impairment [adjusted OR, 3.59; 95% CI, 1.14, 11.25] (Table 5).

**Table 5:** Summary of studies assessing diet/nutrition and other factors associated with an ageing outcome quantitatively

Studies	Exposures of interest	Outcomes assessed	Study design	Risk ratio (95% Cl)	Analytic population
**Diet or nutrition**					
Fu et al. 2021 [41]	GnRI	Longevity (reached 100 years or not)	Cross-sectional	A higher vs. lower GnRI: OR (95% CI): 0.90 (0.88, 0.92)	Those aged 80–99 years
Li et al. 2021 [27]	DDS	Depression	Cross-sectional	Low vs. high: OR (95% CI): 2.24 (1.01–4.96)	Centenarians had a higher proportion of DDS than non-centenarians
Li et al. 2021 [27]	DDS	Anxiety	Cross-sectional	Low vs high: OR (95% CI): 1.56 (0.64, 3.82)	Among centenarians and non-centenarians
Lv et al. 2020 [50]	DDS	Mortality	Prospective cohort	Per unit increase: OR (95% CI): 0.93 (0.92, 0.94)	Comparison made among centenarians
Lv et al. 2020 [50]	DDS	Mortality	Prospective cohort	DDS 4, 5, ≥ 6 vs. < 2: HR (95% CI): 0.69 (0.66, 0.72); 0.65 (0.62, 0.68); 0.56 (0.53, 0.58)	Among the centenarians and non-centenarians
Montesanto et al. 2019 [38]	Malnutrition	Mortality	Prospective cohort	No vs. yes, Kaplan–Meier survival curves show longevity until 100 years + (*p* < 0.001)	All age groups combined
Wu et al. 2017 [28]	Salt preference	ADL impairment	Cross-sectional	Salt preference: OR (95% CI): 3.59 (1.14, 11.25)	All centenarians
Wu et al. 2017 [28]	A good diet habit*	ADL impairment	Cross-sectional	Good diet (yes vs. no): OR (95% CI): 0.49 (0.32, 0.74)	All centenarians
Body weight					
Fu et al. 2021 [41]	Abdominal obesity	Longevity	Cross-sectional	Abdominal obesity (%), per unit increase, OR (95% CI): 0.72 (0.52–0.996)	All centenarians
Fu et al. 2021 [41]	Body weight	Longevity	Cross-sectional	Body weight, per kg increase, OR (95% CI): 0.92 (0.90,0.94)	All centenarians
Lv et al. 2018 [51]	BMI	ADL impairment	Prospective cohort	Underweight vs. normal weight: (quintile, Q4: normal weight category): Q1 vs. Q4: ORs (95% CI): 1.21 (1.05, 1.39); Q2 vs. Q4: 1.32 (1.14, 1.52); Q3 vs. Q4: 1.14 (0.99, 1.32); Q5 (overweight) vs. Q4: 1.07 (0.91–1.26)	All centenarians
Lv et al. 2018 [51]	BMI	ADL impairment	Prospective cohort	Overweight vs. normal weight: OR (95% CI): 0.84 (0.78–0.91)	All age groups combined
**Other lifestyle factors**					
Hao et al. 2019 [23]	Sleep satisfaction	Life satisfaction	Cross-sectional	Sleep satisfaction, beta = 2.999; SE = .432 (*p* < .001)	All centenarians
Hao et al. 2019 [23]	Smoking status	Life satisfaction	Cross-sectional	Smoking, beta = − 0.099, SE = 0.858 (*p* = 0.908)	All centenarians
Montesanto et al. 2019 [38]	Smoking status	Mortality	Prospective cohort	Yes vs. no: HR (95% CI): 1.79 (0.87, 3.70)	Among centenarians and non-centenarians
Wu et al. 2017 [28]	Smoking status	ADL impairment	Cross-sectional	No vs. yes: OR (95% CI): 0.52 (0.22, 1.23)	All centenarians
Montesanto et al. [38]	Hand grip (≥ 10 kg vs. < 10 kg for females; ≥ 20 kg vs. < 20 kg for males)	Mortality	Prospective cohort	Kaplan–Meier survival curves show longevity until 100 years + (*p* < 0.001)	All age groups combined

^*^Good diet habits were defined as avoiding sweet, fatty, high-cholesterol food and salted food [28]

^**^All ORs or HRs were adjusted for confounders

*ADL*, activities of daily living; *BMI*, body mass index; *CI*, confidence interval; *DDS*, dietary diversity score; *GnRI*, geriatric nutrition risk index; *HR*, hazard ratio; OR, odds ratio; *SE*, standard error

The other significant risk factor among centenarians was body weight. Being overweight but not obese appeared to reduce the risk of impairment in ADL [Overweight vs. Normal weight: adjusted OR:0.84; 95% CI, 0.78,0.91) [51]. Underweight, however, increased the risk of ADL impairment by 21–32% [comparing underweight (quintile 1 and 2) with normal weight (quintile 4) among centenarians], while overweight may decrease the risk of ADL impairment [51]. The other cross-sectional analysis suggests that overweight [per kg increase, OR, 0.92; 95% CI, 0.90,0.94] and abdominal obesity [per unit increase, OR, 0.72; 95% CI, 0.52, 0.996] reduced the likelihood of reaching 100+ among those 80–99 years [41]. However, it is unclear whether these results are adjusted for confounders.

Other significant factors included sleep satisfaction associated with life satisfaction [23] and hand grip strength, which reduced the risk of death [38]. However, smoking status did not appear to be linked with the outcomes assessed [38].

## Discussion

In this systematic review, we examined lifestyle and health practices related to healthy ageing among centenarians (*n* = 32 studies) and near-centenarians (individuals aged 95 + , *n* = 2 studies [38, 39]) across the globe. Several significant trends emerged from our pooled analysis or narrative synthesis, highlighting that diet, body weight, sleep and rural lifestyles may contribute to extreme longevity.

### Diet and nutrition

Our narrative review underscores the importance of maintaining a diverse diet with controlled salt intake as an essential dietary factor in promoting healthy longevity. This finding is consistent with a large body of evidence, such as those illustrating the health benefits of a Mediterranean diet [52] or a greater diet diversity, including regular consumption of milk and grain-based foods [53], to extreme longevity in centenarians. Furthermore, our review suggests that most centenarians preferred a low-salt diet. The only study [27] that mentioned the mean intake of daily sodium of 1648 mg in our review is still within the WHO guideline of < 2 g/day sodium [54]. In other studies, among centenarians, the traditional Okinawan diet contains a daily estimate of 1,113 mg of sodium [55] and the prevalence of hypertension was low (19%) among 73 Italian centenarians [56]. Consistent with this line of evidence among centenarians, multiple studies in the general older populations have demonstrated that high salt diets increased the risks of cognitive decline or dementia [57] and mortality [58, 59] while replacing pure salt with potassium-enriched salt substitute reduced the incidence of cardiovascular events and deaths [60].

Furthermore, the lack of reporting on supplement intake may indicate that supplement use was likely low in this population. A systematic review and meta-analysis have suggested that oral nutritional supplements did not reduce malnutrition or adverse outcomes in frail older adults [61], indicating that the health benefits of supplement intake in improving age-related health outcomes remain unclear.

The relationship between BMI and mortality tends to be U-shaped [62]. Our review suggests that being underweight increases the risk of ADL impairment and mortality, while being overweight may protect against ADL impairment. This finding is consistent among centenarians alone and those aged in their 80 s or 90 s [51]. This evidence suggests that older adults who are underweight or obese, as well as those who had weight loss over 5–10% [63], should be monitored and intervened before adverse outcomes occur. Additionally, ongoing research suggests the benefits of time-restricted eating in metabolic health and human lifespan [64, 65].

### Rural living styles

This review highlights a noteworthy finding: over 75% of centenarians lived in rural areas, suggesting that rural lifestyles may contribute significantly to prolonged health and longevity. After excluding three studies reporting over 90% of centenarians in rural areas [28, 45, 66], our sensitivity analysis [24, 66, 67] yielded a pooled estimate of 59% (22–96%) of the included centenarians or near centenarians who lived in rural areas. The high prevalence of centenarians residing in rural areas aligns with the observation that Blue Zones, regions with a high concentration of centenarians worldwide, are predominantly situated in remote islands [68]. It is plausible that many studies included in this review targeted areas with higher centenarian concentrations. However, the overall distribution of centenarians corresponds to the general population globally [69, 70], with exceptions in specific locations [70]. Given the predominant urban residence, enhancing green spaces, tree canopy and public parks to encourage rural lifestyles may boost life expectancy [71] and postpone epigenetic ageing [72].

### Sleep satisfaction

Sleep satisfaction, encompassing sleep efficiency, latency, duration and wake after sleep onset, indicates sleep quality [73]. In a study of three European cohorts, individuals without sleep disturbance compared to those with severe sleep disturbance were projected to live six additional years in good health and three more years without chronic diseases between the age of 50 and 75 [74]. Moreover, sleep satisfaction was found to modulate the link between occupational stress and metabolic syndrome or BMI [74], while both long (> 8 h) and short (< 7 h) sleep durations were associated with an increased risk of death [75]. This evidence underscores the independent role of sleep satisfaction in promoting longevity.

### Medication use

Finally, we anticipated that many centenarians or near-centenarians would take medications to manage chronic health conditions. However, comparing our results to people aged 75 or over (2/3 took five or more medicines) [76] and those living in nursing facilities (95% took 5+ medicines) [77], the prevalence of polypharmacy in this review is low. Consistent with our finding of an average of 4.6 medications, the Epichron study (2011–2015) suggests an average of 4.9 medications for centenarians while over 6.7 medications for those aged 80 + [78]. Hence, our pooled results may otherwise support the low disease prevalence among centenarians/near-centenarians, leading to a lower average number of medications [6, 8–10]. Like other observational studies, we recognised that older people may have underreported or underdiagnoses of chronic conditions or medication use.

### Strengths and limitations

In contrast to previous centenarian-focused reviews [52, 55, 79, 80], our study is the first to employ a systematic review methodology across multiple databases to investigate diet, medication use and various lifestyles and health practices contributing to successful ageing among individuals with extreme longevity. Pooled summary results on demographic characteristics, lifestyle factors, and chronic health conditions were presented whenever possible. Due to substantial variability (as indicated by high *I*^2^ in the metaprop procedure), a narrative synthesis was chosen to summarise findings on dietary or other factors associated with health outcomes. This approach aligns with previous reviews [52, 55, 80]. While we cannot conduct a meta-analysis for pooled effect estimates of risk ratios due to this limitation, our results corroborate those from earlier reviews and high-quality individual studies on centenarians. Future research should prioritise specific research questions and aspects of extreme longevity to foster the development of standardised and rigorous methodologies for empirical evidence across diverse centenarian populations.

Many studies in this review adopted a cross-sectional design, posing challenges in establishing causal relationships. Additionally, a majority focused on relatively healthy centenarians, making comparisons between exposed and unexposed groups within this population less conclusive due to the absence of a non-centenarian comparator group. Ideally, including a mixed population from the same birth cohort would offer more insightful results [53]. As noted in earlier reviews, reporting biases in dietary recalls from centenarians raises concerns about the accuracy of reflecting lifetime dietary changes [53]. However, an Italian study with 25 centenarians found consistent patterns in tracking lifestyle changes over time [81]. Given the high variations and challenges in reporting lifestyle practices, future studies should leverage diverse data sources, including digital technology (e.g., the low prevalence of physical activity in this review indicates the challenge of self-reporting of physical activity and its different forms), nutritional biomarkers and microbiome analysis, to capture changes in this extreme ageing population. Due to these reasons, it is prudent to exercise caution when interpreting the findings presented in this review.

## Conclusion

This systematic review, centred on healthy ageing and extreme longevity, underscores the critical importance of a nutritionally balanced and diverse diet, controlled salt intake and body weight maintenance in mitigating mortality risks and physical functional decline. Moreover, the influence of rural lifestyles and sleep satisfaction warrants further investigation, given their potential roles in facilitating successful ageing. These insights offer valuable guidance for enhancing healthcare practices and crafting lifestyle-based medicine approaches to promote the high quality of ageing life amid the expanding older populations worldwide.

## Supplementary Information

Below is the link to the electronic supplementary material.

Supplementary file1 (DOCX 273 KB)
