# Supplementary material for: A systematic review of diet and medication use among centenarians and near-centenarians worldwide
Source: GeroScience. 2024 Jul 5;46(6):6625–39. doi: 10.1007/s11357-024-01247-4 (PMC11493889; doi:10.1007/s11357-024-01247-4)
Supplement: Supplementary file 1 — Supplementary file1 (DOCX 273 KB) [file 11357_2024_1247_MOESM1_ESM.docx]

**Dai et al.** A systematic review of diet and medication use among centenarians and near-centenarians worldwide

**Supplementary materials**

**S1. Search strategies**

Medline (via Ovid)

| 1 | Centenarian*.mp. |
| --- | --- |
| 2 | Oldest-old.mp. |
| 3 | (100 years old and over).mp. |
| 4 | Aged 100+.mp. |
| 5 | Longevity.mp. |
| 6 | 1 or 2 or 3 or 4 or 5 |
| 7 | Healthy ageing.mp. or exp healthy ageing/ |
| 8 | Healthy aging.mp. or exp healthy aging/ |
| 9 | Successful ageing.mp. or exp successful ageing/ |
| 10 | Successful aging.mp. or exp successful aging/ |
| 11 | 7 or 8 or 9 or 10 |
| 12 | 6 and 11 |
| 13 | limit 12 to (English language and humans and yr= “2000 -Current”) |

CINAHL

| S1 TI ( “Centenarian*” or “Oldest-old” or “100 years old and over” or “Aged 100+” or “Longevity” ) OR AB ( “Centenarian*” or “Oldest-old” or “100 years old and over” or “Aged 100+” or “Longevity” ) OR MH ““Centenarian*” OR MH (““100 years old and over” ) OR MH ““Longevity” |
| --- |
| S2 TI ““Healthy ageing”” OR AB ““Healthy ageing”” OR TI ““Successful ageing”” OR AB ““Successful ageing”” OR TI ““Healthy aging”” OR AB ““Healthy aging”” OR TI ““Successful aging”” OR AB ““Successful aging”” OR MH ( healthy ageing or healthy aging or aging well or ageing well ) OR MH ( successful ageing or successful aging ) |
| S1 & S2 |

Scopus

( TITLE-ABS-KEY ( centenarian* OR oldest-old OR ( 100 years AND old AND over ) OR aged AND 100+ OR longevity ) AND TITLE-ABS-KEY ( healthy AND ageing OR healthy AND aging OR successful AND ageing OR successful AND aging ) ) AND ( LIMIT-TO ( PUBYEAR , 2023 ) OR LIMIT-TO ( PUBYEAR , 2022 ) OR LIMIT-TO ( PUBYEAR , 2021 ) OR LIMIT-TO ( PUBYEAR , 2020 ) OR LIMIT-TO ( PUBYEAR , 2019 ) OR LIMIT-TO ( PUBYEAR , 2018 ) OR LIMIT-TO ( PUBYEAR , 2017 ) OR LIMIT-TO ( PUBYEAR , 2016 ) OR LIMIT-TO ( PUBYEAR , 2015 ) OR LIMIT-TO ( PUBYEAR , 2014 ) OR LIMIT-TO ( PUBYEAR , 2013 ) OR LIMIT-TO ( PUBYEAR , 2012 ) OR LIMIT-TO ( PUBYEAR , 2008 ) OR LIMIT-TO ( PUBYEAR , 2007 ) OR LIMIT-TO ( PUBYEAR , 2006 ) OR LIMIT-TO ( PUBYEAR , 2005 ) OR LIMIT-TO ( PUBYEAR , 2004 ) OR LIMIT-TO ( PUBYEAR , 2003 ) OR LIMIT-TO ( PUBYEAR , 2002 ) OR LIMIT-TO ( PUBYEAR , 2001 ) OR LIMIT-TO ( PUBYEAR , 2000 ) ) AND ( LIMIT-TO ( DOCTYPE , ““ar””) OR LIMIT-TO ( DOCTYPE , ““no””) ) AND ( LIMIT-TO ( LANGUAGE , ““English””) )AND ( LIMIT-TO ( EXACTKEYWORD , ““Human””) )

**Table s1.** Inclusion and exclusion criteria for the 34 included studies

| **Inclusion criteria** | **Exclusion criteria** |
| --- | --- |
| **Study design:** Quantitative epidemiological studies, including cohort studies, case-control studies, cross-sectional studies.  Intervention studies or randomised control trials are less likely to be available considering such an extremely old age, but we would include them if available.  **Age:** those aged 100 years or above, or the mean or median age of the study sample would be 95 years or above. We included studies among centenarians alone or comparing centenarians with non-centenarians.   **Study focus:** studies focusing on healthy ageing or successful ageing and reported target population’s diet, food intake, nutrition status (including nutrition markers, body composition, and anthropometrics), supplement use, and medication use.  **Published language:** English  **Publication period:** 2000-2022 (Search period: 1 January 2000- 10 December 2022) | **Study design:** reviews, systematic reviews, opinions, single case reports, dissertations, conference abstracts or proceedings, book chapters, or editorials.   **Study exposures/outcomes:** no exposures of interest or primary outcomes included, or those without any ageing outcomes.   **Study focus:** those on genes or genetic profiles.   **Non-human studies:** studies using animals or cellular models to study ageing in any aspect. |

**Table s2.** Characteristics of included studies (n=34)

| **Study ID** | **Country** | **Study design** | **Follow-up time** | **Sample size of the whole study** | **No. of centenarians or near centenarians** | **Centenarian identification** | **Mean (SD) or median age** | **No. of non-centenarians)** | **Mean (SD) or median age** | **Comparator Group** | **Exposure of interest** | **Outcome of interest** |
| --- | --- | --- | --- | --- | --- | --- | --- | --- | --- | --- | --- | --- |
| Alis et al. 2016 [1] | Italy | Cross-sectional study |  | 127 | 81 | Birth dates on identity cards | Range: 100-104 years | 46 | Range 70-80 years | Among centenarians and non-centenarians | Blood levels of Se and Cu | Successful ageing (reached 100 years or above) |
| Andersen-Ranberg et al. 2001 [2] | Denmark | Cross-sectional study |  | 207 | 207 (162 F, 45 M) | Danish Civil Registration System | Not clear | 0 | N/A | Male vs Female centenarians | Nutritional factors and medication use | NR (descriptive analysis) |
| Basile et al. 2003 [3] | Italy | Cross-sectional study |  | 48 | 16 (4 men and 12 women) | Unclear | Range: 100-105 | 32 | Group A: 54.06±6.49  Group B: 73.69±6.13 | Among centenarians and non-centenarians | Retinol in each age group | Alpha-tocopherol in each age group |
| Bucci et al. 2014 [4] | Italy | Cohort study | 5 years | 116 | 116 (23 M, 93F) | Government authorities | mean: 100.7 years, range: 99-111 years. | 0 | N/A | All centenarians | Immune parameters | Survival time |
| Cai et al. 2022 [5] | China | Cross-sectional study |  | 61 | 30 “healthy” centenarians | Identity cards | 103±3 | 31 | 63±3 | Among centenarians and non-centenarians | Dietary fibre | Short-chain fatty acids |
| Croize-Pourcelet et al. 2022 [6] | France | Cross-sectional study |  | 22 | 22 (17 F, 5 M) | Unclear | Median: 101 IQR: 100-101 | 0 | N/A | All centenarians (Nursing home vs Private housing) | Nutritional status and medications | Descriptive analysis by living place |
| Darviri et al. 2008 [7] | Portugal | Cross-sectional study |  | 47 | 47 (33 F, 14 M) | *municipal or voting registries* | Age range (min/max): 100/110, Mean(±SD): 101.7(2.2), Median: 101 | 0 | N/A | Male vs Female centenarians | Anthropometric and body composition | Anthropometric and body composition (descriptive analysis) by gender |
| Fastame et al. 2022 [8] | Greece | Cross-sectional study |  | 57 | 17 Participants over 99 years.   Long-lived group; 28 (15 males, 13 females) | Unclear | Long-lived group; Mean (SD): 97.6 years (7.9), Age range: 90-105 years | 29 | Mean: 83.9±2.7  Range: 80-89 years | Among centenarians and octogenarians | Anthropometric measures | Health status (descriptive analysis) |
| Forte et al. 2014[9] | Italy | Cross-sectional study |  | 164 | 64 | Unclear | Mean: 101±1;Range: 100-104 | 76 nonagenarians  24 controls | Nonagenarian mean: 89±6.3, range: 80-99 years  Controls mean: 61.2±1.1, range: 60-63 years | Among centenarians, nonagenarians, and those aged 60-63 years | Fruits, vegetables, carbohydrates, and proteins | Descriptive analysis by age group |
| Fu et al. 2020 [10] | China | Cross-sectional study |  | 750 | 750 (608 F, 142 M) | National Civil Registry | 102 | 0 | N/A | All centenarians (eGFR<60ml/min vs eGFR≥60ml/min) | Plasma mineral or vitamin levels | eGFR level |
| Fu et al. 2021 [11] | China | Cross-sectional study |  | 737 | 500 | National Civil Registry | range: 100+:  Mean (SD): 103±2.9 | 237 | Mean: 86±4.9  Range: 80-99 years | Among centenarians and non-centenarians | Nutritional status, sex hormones, bone turnover markers | Being a centenarian, geriatric nutrition index, and abdominal obesity |
| Hagberg et al. 2008 [12] | Sweeden | Cohort study | 11 years | 100 | 100 (82 F, 18 M) | National register | 100-111 | 0 | N/A | All centenarians | Various factors, including food intake, body composition, functional outcomes; number of drugs, and family longevity | Survival after 100 years |
| Hai et al. 2022 [13] | China | Cross-sectional study |  | 586 | 253 | Birth dates on identity cards | Median: 100.0 Range: 99.0-109.0 | 333 (Offspring=217, Offspring’s spouses=116) | Offspring median: 70 years;  Offspring’s spouses median: 69 years | Among centenarians, centenarian offspring, and offspring spouses | Centenarians vs. Offspring spouses | Prevalence of disease or nutritional indicators |
| Hao et al. 2016 [14] | China | Cross-sectional study |  | 379 | 255 | Unclear | 100-103: 166 104-107: 63 Greater than or equal to 108: 26 | 124 | Not specified | Among centenarians, centenarian children, and centenarian grandchildren | Age group | Hair level of Cr, Fe, Na, Ni, P, Pb, Zn, Ca, Cr, Mg, Mn, P, Se, and Sr.” |
| Hao et al. 2019 [15] | China | Cross-sectional study |  | 273 | 223 (45 M, 178 F) | Registry of Bureau of Civil Affairs | 100-103  104-107 108-112 Average age: 103.30(3.07) | 50 | Not specified | Male vs Female centenarians | Anthropometric measures | Descriptive analysis by gender |
| He et al. 2018 [16] | China | Cross-sectional study |  | 990 | 990 (179 M, 811 F) | Registry of the Department of Civil Affairs of Hainan Province | Mean: 102.85±2.76 Range: 100-115 | 0 | N/A | Male vs Female centenarians | Trace elements level | Descriptive analysis and correlation analysis |
| Li et al. 2012 [17] | China | Cross-sectional study |  | 78 | 78 (68 female, 10 male) | Unclear | Male mean: 102.20±1.23 Male median: 102.50 Female mean: 102.69±2.17 Female median: 102 | 0 | N/A | Male vs Female centenarians | Demographics or disease by sex | N/A |
| Li et al. 2021 [18] | China | Cross-sectional study |  | 288 | 38 | Birth date on the ID card | Median: 101 IQR: 100-102 Range: 100-110 | 250 | Median: 71 years | Among centenarians and non-centenarians | Dietary diversity score | Depression and anxiety |
| Li et al. 2022 [19] | China | Cross-sectional study |  | 117 | 27 | Population information provided by local government authorities | Range: 100-118 Mean: 103.41±4.14 | 90 | LRN group mean: 93±2.17, range: 90-99  LRE group mean: 70.08±8.24, range: 60-89  NLRE group mean: 71.84±7.59, range: 60-89 | Among centenarians (LRC group) and non-centenarians (LRN group) | Age groups | Various levels of metabolites |
| Lv et al. 2018 [20] | China | Cohort study | 6 years | 16022 | 3531 | Unclear | Age range:100 years or older | 12491 | Octogenarians range: 80-89 years  Nonagenarians range: 90-99 years | All combined (Comparator groups by BMI quintiles) | BMI | ADL disability |
| Lv et al. 2020 [21] | China | Cohort study | 6 years | 28790 | 8908 | Unclear | Not specified | 9957 | Octogenarians range: 80-89 years  Nonagenarians range: 90-99 years | Among centenarians and non-centenarians | Dietary diversity scores | Mortality |
| Lv et al. 2021[22] | China | Cross sectional |  | 2501 | 386 | Unclear | Not specified | 2115 | Younger elderly mean: 72.44±3.95  Octogenarian mean: 84.28±2.83  Nonagenarian mean: 93.34±2.71 | Among centenarians and non-centenarians | Metal levels such as arsenic by chronic kidney disease status, selenium by weight status, and molybdenum by anaemia status | Metal levels by condition |
| Magri et al. 2002 [23] | Italy | Cross-sectional study |  | 68 | 24 (24 F) | Unclear | Range: 100-106 Mean: 101.6±0.38 | 44 | 71-93 years mean: 84.75±1.25  22-33 years mean: 27.84±0.66 | Among centenarians and non-centenarians (71-93 years & 22-33 years) | Thyroid hormone levels, T3/T4 | Anthropometric parameters |
| Mecocci et al. 2000 [24] | Italy | Cross-sectional study |  | 107 | 32 | Free-living subjects | Mean: 100.56±0.7 | 75 | Range: <99 | Among centenarians and non-centenarians (81-99 years, 61-80 years, <60 years) | Age by centenarian status | Plasma Antioxidants |
| Montesanto et al. 2019[25] | Italy | Cohort study | 7 years | 355 | 355 (centenarians not separated from nonagenarians) | Department of Civil Affairs of Hainan Province | > 90 years old, mean (SD): 96.64 (3.313) | N/A | N/A | All combined | Cholesterol, diabetes, hypertension, smoking, and CVD risk factors | Mortality |
| daSilva et al. 2016 [26] | Italy | Cross-sectional study |  | 252 | 252 | Unclear | Mean: 100.26 (1.99) Age range: 97-109 years | 0 | N/A | Male vs Female centenarians | Anthropometrics (BMI, waist: hip ratio) | Body fat |
| Savarino et al. 2001[27] | Italy | Cross-sectional study |  | 152 | 42 (25 females and 17 males)- part of group A in the study. | Unclear | 101-107, 101.5(1.3), median: 101.0 | 110 (Comparison between 91-110 years (n=90) and 61-90 years (n=46)) | 60-90 years mean:71.2±9.2, median:70.0 years  90-100 years mean: 94.4±2.4, median: 95.0 years | 91-110 years vs 60-90 years | age | Zn/Se level |
| Schmidt et al. 2018 [28] | Germany | Cohort study | 6 years | 1398 | 398 | Information based on healthcare insurance | Range: 100+ | 1000 | 80+ years (n=500)  90+ years (n=500) | Among centenarians, 90+ years, and 80+ years | Sex, age group, comorbidities, and medication prescriptions | Heart failure |
| Stathakos et al. 2005 [29] | Greece | Cross-sectional study |  | 489 | 489 (113 M, 376 F) | National insurance and voting registers | Male: Median: 101.0 Mean (SD): 101.6 (1.9) Female: Median:101.0 Mean (SD): 101.8 (2.5) | 0 | N/A | Male vs Female centenarians |  | Various health conditions (descriptive analyses) |
| Takayama et al. 2007 [30] | Japan | Cross-sectional study |  | 302 | 302 (65 M, 237 F) | Basic residential register at the administrative institution of each of 23 wards of the Tokyo metropolitan area. | Mean: 101.2±1.8 Median: 100 IQR: 102-100 | 0 | N/A | Male vs Female centenarians | medicine by sex; disease by sex or Barthel Index (BI) or Clinical Dementia Rating Scores | Descriptive analysis of medicine and diseases |
| Tigani et al. 2012 [31] | Greece | Cross-sectional study |  | 400 | 400 | Birth dates on official identification cards | Age range: 100-109 Mean: 101.85 | 0 | N/A | All centenarians | Various factors, including sociodemographic, disease-related,  lifestyle and psychosocial variables | Self-reported health |
| Wong et al. 2019 [32] | Australia | Cross-sectional study |  | 100 | 20 (95+ years) | Unclear | Mean: 96.6±1.4 | 80 | 79.1±2.9 | All combined (Comparator groups by lipid profiles) | Age, BMI, and sex | Lipid levels |
| Wu et al. 2017 [33] | China | Cross-sectional study |  | 564 | 564 (93 M, 471 F) | Examining the hukou and current residences | 100-105 years and 106-110 years | 0 | N/A | All centenarians (Comparator groups: No ADL disability vs ADL disability) | Diet, and other lifestyle factors | ADL disability |
| Zhang et al. 2020 [34] | China | Cross-sectional study |  | 95 | 95 (83 F, 12 M) | Unclear | 100-103 (60 centenarians), 104-107 (32 centenarians), over 108 years (3 centenarians) | 0 | N/A | All centenarians (Grouped by age: 1001-103 years, 104-107 years, and 108+ years) | Dietary and lifestyle factors (yes/no) | Serum levels of Se |

**Table s3. Risk of bias assessment for each included study**

| **Study ID** | **All study groups derived  from similar source/reference populations?** | **Attrition not significantly  different across study groups?** | **Is the measure of exposure valid?** | **Is the measure of outcome valid?** | **Potential confounders identified (e.g., comorbidities, multicomponent interventions, etc.)?** | **Is statistical adjustment for potential confounders done?** | **Funding source(s) disclosed** | **Conflict of interest identified** |
| --- | --- | --- | --- | --- | --- | --- | --- | --- |
| Alis et al. 2016 [1] | Not clear | Not applicable | Not clear | High (Secure records or directly measured) | Yes | Yes | Yes | No |
| Andersen-Ranberg et al. 2001 [2] | No | Not applicable | High (Secure records or directly measured) | High (Secure records or directly measured) | No | No | No | Missing |
| Basile et al. 2003 [3] | Yes | No | High (Secure records or directly measured) | High (Secure records or directly measured) | No | No | Unclear | No |
| Bucci et al. 2014[4] | Yes | Not applicable | High (Secure records or directly measured) | High (Secure records or directly measured) | NR | NR | Yes | No |
| Cai et al. 2022 [5] | Yes | No | High (Secure records or directly measured) | High (Secure records or directly measured) | No | No | Yes | No |
| Croize-Pourcelet et al. 2022 [6] | Yes | Not applicable | High (Secure records or directly measured) | High (Secure records or directly measured) | No | No | Yes | No |
| Darviri et al. 2008 [7] | Yes | Not applicable | High (Secure records or directly measured) | High (Secure records or directly measured) | NR | NR | Yes | No |
| Fastame et al. 2022 [8] | Yes | Not applicable | Low (Self-reported) | Low (Self-reported) | Yes | Yes | Yes | No |
| Forte et al. 2014 [9] | Yes | Not applicable | High (Secure records or directly measured) | High (Secure records or directly measured) | NR | NR | Yes | No |
| Fu et al. 2020 [10] | Yes | Not applicable | High (Secure records or directly measured) | High (Secure records or directly measured) | Yes | Yes | Yes | No |
| Fu et al. 2021 [11] | Yes | Not applicable | High (Secure records or directly measured) | High (Secure records or directly measured) | No | No | Yes | Missing |
| Hagberg et al. 2008 [12] | Yes |  | High (Secure records or directly measured) | High (Secure records or directly measured) | No | No | Unclear | No |
| Hai et al. 2022 [13] | Yes | Not applicable | High (Secure records or directly measured) | High (Secure records or directly measured) | Yes | Yes | Yes | Missing |
| Hao et al. 2016 [14] | Yes | Not applicable | High (Secure records or directly measured) | High (Secure records or directly measured) | Yes | Yes | Unclear | No |
| Hao et al. 2019 [15] | Yes | Not applicable | Low (Self-reported) | Low (Self-reported) | Yes | Yes | Yes | No |
| He et al. 2018 [16] | Yes | Yes | High (Secure records or directly measured) | Low (Self-reported) | Yes | No | Yes | Missing |
| Li et al. 2012 [17] | Yes | Not applicable | High (Secure records or directly measured) | High (Secure records or directly measured) | No | No | Unclear | No |
| Li et al. 2021 [18] | Yes | Not applicable | High (Secure records or directly measured) | High (Secure records or directly measured) | Yes | Yes | Yes | No |
| Li et al. 2022 [19] | Yes | Not applicable | High (Secure records or directly measured) | High (Secure records or directly measured) | Yes | Yes | Yes | No |
| Lv et al. 2018 [20] | Yes |  | High (Secure records or directly measured) | High (Secure records or directly measured) | Yes | Yes | Yes | Missing |
| Lv et al. 2020 [21] | Yes | No | Low (Self-reported) | High (Secure records or directly measured) | Yes | Yes | Yes | No |
| Lv et al. 2021[22] | Yes | Not applicable | High (Secure records or directly measured) | High (Secure records or directly measured) | Yes | Yes | Yes | Missing |
| Magri et al. 2002 [23] | Yes | Not applicable | High (Secure records or directly measured) | High (Secure records or directly measured) | No | No | No | Missing |
| Mecocci et al. 2000 [24] | Yes | Not applicable | High (Secure records or directly measured) | High (Secure records or directly measured) | NR | NR | Unclear | No |
| Montesanto et al. 2019 [25] | Yes | Not applicable | High (Secure records or directly measured) | High (Secure records or directly measured) | Yes | Yes | Yes | Missing |
| daSilva et al. 2016 [26] | Yes | Not applicable | High (Secure records or directly measured) | High (Secure records or directly measured) | No | No | No | No |
| Savarino et al. 2001 [27] | No | Not applicable | High (Secure records or directly measured) | High (Secure records or directly measured) | No | No | Yes | Missing |
| Schmidt et al. 2018 [28] | Yes | Not applicable | High (Secure records or directly measured) | High (Secure records or directly measured) | Yes | Yes | Yes | Yes |
| Stathakos et al. 2005 [29] | Yes | Not applicable | Low (Self-reported) | Low (Self-reported) | No | No | No | Missing |
| Takayama et al. 2007 [30] | Yes | No | High (Secure records or directly measured) | Low (Self-reported) | NR | No | Yes | Missing |
| Tigani et al. 2012 [31] | Yes | Not applicable | Low (Self-reported) | Low (Self-reported) | Yes | Yes | Yes | No |
| Wong et al. 2019 [32] | Yes | Not applicable | High (Secure records or directly measured) | High (Secure records or directly measured) | Yes | Yes |  | No |
| Wu et al. 2017 [33] | Yes | Not applicable | Low (Self-reported) | Low (Self-reported) | Yes | Yes | Yes | No |
| Zhang et al. 2020 [34] | Yes | Not applicable | Low (Self-reported) | High (Secure records or directly measured) | No | No | Yes | No |

**Supplementary Figures**: Forrest plots

**Figure s1.** Proportion of female centenarians/near centenarians

**Figure s2.** Proportion of education level

1. Below high school

b. High school or above

**Figure s3.** Proportion of living arrangements

a. Living alone

1. Living with others

1. Living in nursing facilities

**Figure s4.** Regionality

1. Urban area

1. Rural area

**Figure s5.** Smoking status

1. Former smokers

1. Current smokers

**Figure s6**. Drinking status

1. Former drinkers

1. Daily drinkers

**Figure s7.** Physical activity

**Figure s8.** Sleep satisfaction

**Figure s9.** Weight status

1. Underweight

1. Normal weight

1. Overweight

1. Obese

**Figure s10.** Prevalence of common medications

1. Antihypertensives

1. CVD medications

**Figure s11.** Prevalence of common health conditions

1. Basic ADL

1. Hypertension
2. Dementia or cognitive impairment

1. Type 2 diabetes

**References**

1. Alis R, Santos-Lozano A, Sanchis-Gomar F, Pareja-Galeano H, Fiuza-Luces C, Garatachea N, et al. Trace elements levels in centenarian 'dodgers'. Journal of trace elements in medicine and biology : organ of the Society for Minerals and Trace Elements (GMS). 2016;35(b7q, 9508274):103-6.

2. Andersen-Ranberg K, Schroll M, Jeune B. Healthy centenarians do not exist, but autonomous centenarians do: A population-based study of morbidity among danish centenarians. Journal of the American Geriatrics Society. 2001;49(7):900-8.

3. Basile G, Gangemi S, Lo Balbo C, Mento A, Nicita-Mauro C, Crisafulli G, et al. Correlation between serum retinol and α-tocopherol levels in centenarians. Journal of Nutritional Science and Vitaminology. 2003;49(4):287-8.

4. Bucci L, Ostan R, Giampieri E, Cevenini E, Pini E, Scurti M, et al. Immune parameters identify Italian centenarians with a longer five-year survival independent of their health and functional status. Experimental gerontology. 2014;54(epq, 0047061):14-20.

5. Cai D, Zhao Z, Zhao L, Dong Y, Wang L, Zhao S, et al. The Age-Accompanied and Diet-Associated Remodeling of the Phospholipid, Amino Acid, and SCFA Metabolism of Healthy Centenarians from a Chinese Longevous Region: A Window into Exceptional Longevity. Nutrients. 2022;14(20).

6. Croize-Pourcelet C, Nouguerede E, Rey D, Daumas A, Gentile G, Villani P, et al. Geriatric syndromes in a centenarians population. Aging clinical and experimental research. 2022;34(12):3131-6.

7. Darviri C, Demakakos P, Charizani F, Tigani X, Tsiou C, Chalamandaris AG, et al. Assessment of the health status of Greek centenarians. Archives of Gerontology and Geriatrics. 2008;46(1):67-78.

8. Fastame MC. Well-being, food habits, and lifestyle for longevity. Preliminary evidence from the sardinian centenarians and long-lived people of the Blue Zone. Psychology, health & medicine. 2022;27(3):728-33.

9. Forte G, Deiana M, Pasella S, Baralla A, Occhineri P, Mura I, et al. Metals in plasma of nonagenarians and centenarians living in a key area of longevity. Experimental gerontology. 2014;60(epq, 0047061):197-206.

10. Fu S, Yu H, Li Y, Lv F, Deng J, Zhang F, et al. Multiple Measures of Mineral Metabolism Were Associated With Renal Function in Chinese Centenarians: A Cross-Sectional Study. Front Med (Lausanne). 2020;7:120.

11. Fu S, Ping P, Li Y, Li B, Zhao Y, Yao Y, et al. Centenarian longevity had inverse relationships with nutritional status and abdominal obesity and positive relationships with sex hormones and bone turnover in the oldest females. Journal of translational medicine. 2021;19(1):436.

12. Hagberg B, Samuelsson G. Survival after 100 years of age: a multivariate model of exceptional survival in Swedish centenarians. J Gerontol A Biol Sci Med Sci. 2008;63(11):1219-26.

13. Hai PC, Yao DX, Zhao R, Dong C, Saymuah S, Pan YS, et al. BMI, Blood Pressure, and Plasma Lipids among Centenarians and Their Offspring. Evidence-based Complementary and Alternative Medicine. 2022;2022.

14. Hao Z, Li Y, Liu Y, Li H, Wang W, Yu J. Hair elements and healthy aging: a cross-sectional study in Hainan Island, China. Environmental Geochemistry and Health. 2016;38(3):723-35.

15. Hao Z, Chen L, Li Y, Zou X, Li H, Feng Z, et al. Characteristics of centenarians' lifestyles and their contribution to life satisfaction: A case study conducted on Hainan Island. Archives of gerontology and geriatrics. 2019;83(8214379, 7ax):20-7.

16. He Y, Zhao Y, Yao Y, Yang S, Li J, Liu M, et al. Cohort Profile: The China Hainan Centenarian Cohort Study (CHCCS). International Journal of Epidemiology. 2018;47(3):694-5h.

17. Li Y, Zou X, Lv J, Yang L, Li H, Wang W. Trace Elements in Fingernails of Healthy Chinese Centenarians. Biological Trace Element Research. 2012 2012/02/01;145(2):158-65.

18. Li R, Zong Z-Y, Gu X-X, Wang D-N, Dong C, Sun C, et al. Higher dietary diversity as a protective factor against depression among older adults in China: a cross-sectional study. Annals of Palliative Medicine. 2021;11(4):1278-89.

19. Li H, Ren M, Li Q. 1H NMR-Based Metabolomics Reveals the Intrinsic Interaction of Age, Plasma Signature Metabolites, and Nutrient Intake in the Longevity Population in Guangxi, China. Nutrients. 2022;14(12):2539.

20. Lv YB, Yuan JQ, Mao C, Gao X, Yin ZX, Kraus VB, et al. Association of Body Mass Index With Disability in Activities of Daily Living Among Chinese Adults 80 Years of Age or Older. JAMA Netw Open. 2018 Sep 7;1(5):e181915.

21. Lv Y, Kraus VB, Gao X, Yin Z, Zhou J, Mao C, et al. Higher dietary diversity scores and protein-rich food consumption were associated with lower risk of all-cause mortality in the oldest old. Clin Nutr. 2020 Jul;39(7):2246-54.

22. Lv Y, Wei Y, Zhou J, Xue K, Guo Y, Liu Y, et al. Human biomonitoring of toxic and essential metals in younger elderly, octogenarians, nonagenarians and centenarians: Analysis of the Healthy Ageing and Biomarkers Cohort Study (HABCS) in China. Environ Int. 2021 Nov;156:106717.

23. Magri F, Muzzoni B, Cravello L, Fioravanti M, Busconi L, Camozzi D, et al. Thyroid function in physiological aging and in centenarians: possible relationships with some nutritional markers. Metabolism. 2002 Jan;51(1):105-9.

24. Mecocci P, Polidori MC, Troiano L, Cherubini A, Cecchetti R, Pini G, et al. Plasma antioxidants and longevity: a study on healthy centenarians. Free Radic Biol Med. 2000 Apr 15;28(8):1243-8.

25. Montesanto A, Pellegrino D, Geracitano S, La Russa D, Mari V, Garasto S, et al. Cardiovascular risk profiling of long-lived people shows peculiar associations with mortality compared with younger individuals. Geriatr Gerontol Int. 2019 Feb;19(2):165-70.

26. PereiradaSilva AM, A; Valente, A; Gil, Â; Alonso, I; Ribeiro, R; Bicho, M; Gorjão-Clara, J. Body Composition Assessment and Nutritional Status Evaluation in Men and Women Portuguese Centenarians. Journal of Nutrition, Health & Aging. 2016;20(3).

27. Savarino L, Granchi D, Ciapetti G, Cenni E, Ravaglia G, Forti P, et al. Serum concentrations of zinc and selenium in elderly people: results in healthy nonagenarians/centenarians. Exp Gerontol. 2001 Feb;36(2):327-39.

28. Schmidt IM, Kreutz R, Dräger D, Zwillich C, Hörter S, Kuhlmey A, et al. Lower Prescription Rates in Centenarians with Heart Failure and Heart Failure and Kidney Disease Combined: Findings from a Longitudinal Cohort Study of Very Old Patients. Drugs Aging. 2018 Oct;35(10):907-16.

29. Stathakos D, Pratsinis H, Zachos I, Vlahaki I, Gianakopoulou A, Zianni D, et al. Greek centenarians: assessment of functional health status and life-style characteristics. Exp Gerontol. 2005 Jun;40(6):512-8.

30. Takayama M, Hirose N, Arai Y, Gondo Y, Shimizu K, Ebihara Y, et al. Morbidity of Tokyo-area centenarians and its relationship to functional status. J Gerontol A Biol Sci Med Sci. 2007 Jul;62(7):774-82.

31. Tigani X, Artemiadis AK, Alexopoulos EC, Chrousos GP, Darviri C. Self-rated health in centenarians: a nation-wide cross-sectional Greek study. Arch Gerontol Geriatr. 2012 May-Jun;54(3):e342-8.

32. Wong MWK, Braidy N, Pickford R, Vafaee F, Crawford J, Muenchhoff J, et al. Plasma lipidome variation during the second half of the human lifespan is associated with age and sex but minimally with BMI. PLoS One. 2019;14(3):e0214141.

33. Wu T, Lu L, Luo L, Guo Y, Ying L, Tao Q, et al. Factors Associated with Activities of Daily Life Disability among Centenarians in Rural Chongqing, China: A Cross-Sectional Study. Int J Environ Res Public Health. 2017 Nov 9;14(11).

34. Zhang R, Wang L, Li Y, Li H, Xu Y. Distribution Characteristics of Rare Earth Elements and Selenium in Hair of Centenarians Living in China Longevity Region. Biol Trace Elem Res. 2020 Sep;197(1):15-24.
